# Applied screening tests for the detection of superior face recognition

**DOI:** 10.1186/s41235-018-0116-5

**Published:** 2018-06-27

**Authors:** Sarah Bate, Charlie Frowd, Rachel Bennetts, Nabil Hasshim, Ebony Murray, Anna K. Bobak, Harriet Wills, Sarah Richards

**Affiliations:** 1 Department of Psychology, Faculty of Science and Technology, Bournemouth University, Fern Barrow, Poole, UK; 2 School of Psychology, University of Central Lancashire, Preston, UK; 3 School of Biological and Chemical Sciences, Queen Mary, University of London, London, UK

**Keywords:** Face recognition, Individual differences, Super recognisers, Composite face-processing

## Abstract

In recent years there has been growing interest in the identification of people with superior face recognition skills, for both theoretical and applied investigations. These individuals have mostly been identified via their performance on a single attempt at a tightly controlled test of face memory—the long form of the Cambridge Face Memory Test (CFMT+). The consistency of their skills over a range of tests, particularly those replicating more applied policing scenarios, has yet to be examined systematically. The current investigation screened 200 people who believed they have superior face recognition skills, using the CFMT+ and three new, more applied tests (measuring face memory, face matching and composite-face identification in a crowd). Of the sample, 59.5% showed at least some consistency in superior face recognition performance, although only five individuals outperformed controls on overall indices of target-present and target-absent trials. Only one participant outperformed controls on the Crowds test, suggesting that some applied face recognition tasks require very specific skills. In conclusion, future screening protocols need to be suitably thorough to test for consistency in performance, and to allow different types of superior performer to be detected from the outset. Screening for optimal performers may sometimes need to directly replicate the task in question, taking into account target-present and target-absent performance. Self-selection alone is not a reliable means of identifying those at the top end of the face recognition spectrum.

**Electronic supplementary material:**

The online version of this article (10.1186/s41235-018-0116-5) contains supplementary material, which is available to authorized users.

## Significance

In recent years there has been increasing real-world interest in the identification of people with naturally proficient face recognition skills. Because computerised face recognition systems are yet to replicate the capacity of human perceivers, individuals with superior skills may be particularly useful in policing scenarios. Such tasks may involve matching or identifying faces captured in CCTV footage, or “spotting” wanted perpetrators in a crowd. However, little work has considered the screening tests and protocols that should be used to identify top human performers, and existing real-world and laboratory procedures tend to rely on performance on a single attempt at a test of face memory. The findings of this paper highlight the need for objective screening of all available personnel, without influence of self-selection. Screening protocols should be suitably thorough to allow for detection of different types of superior performer, allowing independent detection of those who are adept at either face memory or face matching. Recruitment of top performers for some very specific face recognition tasks (e.g. those involving artificial facial stimuli) may require direct replication of the task in hand. In sum, these findings call for a review of “super recogniser” screening protocols in real-world settings.

## Background

Increasing work is examining individual differences in face recognition (e.g. Bate, Parris, Haslam, & Kay, 2010; Wilmer, 2017; Yovel, Wilmer, & Duchaine, 2014), with particular interest in people who lie at the two extremes. At the lower end of the spectrum are those with very poor face recognition skills who may have a condition known as “developmental prosopagnosia” (Bate & Cook, 2012; Bennetts, Butcher, Lander, Udale, & Bate, 2015; Burns et al., 2017; Dalrymple & Palermo, 2016; Duchaine & Nakayama, 2006), whereas those at the top end have an extraordinary ability to recognise faces (Bobak, Pampoulov, & Bate, 2016; Russell, Duchaine, & Nakayama, 2009). These so-called “super recognisers” (SRs) are of both theoretical and practical importance: while examination of the cognitive and neural underpinnings of this proficiency can inform our theoretical understanding of the typical and impaired face-processing system (Bate & Tree, 2017; Bennetts, Mole, & Bate, 2017; Bobak, Bennetts, Parris, Jansari, & Bate, 2016; Bobak, Parris, Gregory, Bennetts, & Bate, 2017; Ramon et al., 2016), SRs may also be useful in policing and security settings (Bobak, Dowsett, & Bate, 2016; Bobak, Hancock, & Bate, 2016; Davis, Lander, Evans, & Jansari, 2016; Robertson, Noyes, Dowsett, Jenkins, & Burton, 2016). However, most studies have relied on a single laboratory test of face recognition to identify SRs (for a review see Noyes, Phillips, & O'Toole, 2017) and the consistency of their skills across a larger variety of more applied face recognition tasks has yet to be examined systematically. This is an important issue as the police need to ensure that any officers (or police staff) deployed for specific face recognition tasks are indeed the best candidates for the job.

Clearly, then, a consistent diagnostic approach needs to be implemented by both researchers and their beneficiaries. Most investigations have “confirmed” super recognition in their experimental participants via the long form of the Cambridge Face Memory Test (CFMT+), a test that was initially described in the first published investigation into super recognition (Russell et al., 2009). The CFMT+ is an extended version of the standard Cambridge Face Memory Test (CFMT; Duchaine & Nakayama, 2006), in which participants are required to learn the faces of six individuals, and are tested on 72 triads of faces where they are asked to select one of the target faces. The standard version of the CFMT is a dominant test that is used worldwide to diagnose prosopagnosia (e.g. Bate, Adams, Bennetts, & Line, in press; Bowles et al., 2009; Dalrymple & Palermo, 2016; McKone et al., 2011), and has been shown to have high reliability (Bowles et al., 2009; Wilmer, Germine, Chabris, et al., 2010) and both convergent and divergent validity (Bowles et al., 2009; Dennett et al., 2012; Wilmer, Germine, Chabris, et al., 2010; Wilmer, Germine, Loken, et al., 2010). Alternative versions of the CFMT possess similar properties, indicating that the paradigm provides a reliable assessment of face memory (Bate et al., 2014; McKone et al., 2011; Wilmer, Germine, Loken, et al., 2010). To make the test suitable for the detection of SRs, the CFMT+ follows the identical format of the original CFMT but includes 30 additional, more difficult trials (Russell et al., 2009). Both group-based (Russell et al., 2009) and more conservative case-by-case (e.g. Bobak et al., 2016; Bobak, Dowsett, & Bate, 2016) statistics have been used to identify superior performance on the extended test, suggesting that it is appropriately calibrated for this task.

The latter statistical approach is important when considering the potential for heterogeneity in super recognition, as it allows researchers to examine the consistency of performance in each individual (as opposed to a group as a whole) across tests that tap into different processes. There is a theoretical basis for this assumption of heterogeneity when examining the patterns of presentation that have been observed in those with developmental prosopagnosia. Specifically, while some of these individuals appear to only have difficulties in their memory for faces (e.g. Bate, Haslam, Jansari, & Hodgson, 2009; Lee, Duchaine, Wilson, & Nakayama, 2010; McKone et al., 2011), others also have impairments in the *perception* of facial identity (i.e. when asked to make a judgement on the identity of an individual without placing any demands on memory; Bate et al., 2009; Chatterjee & Nakayama, 2012; Duchaine, Germine, & Nakayama, 2007). Given that this dissociation has also been observed in acquired cases of prosopagnosia (Barton, Press, Keenan, & O’Connor, 2002; De Haan, Young, & Newcombe, 1987, 1991; De Renzi, Faglioni, Grossi, & Nichelli, 1991), and hypotheses that developmental prosopagnosia simply resides at the bottom of a common face recognition spectrum where super recognition lies at the top (Barton & Corrow, 2016; Bate & Tree, 2017), a logical prediction is that some SRs may be proficient at both face memory and face perception, whereas others may have abilities that are restricted to one sub-process. In fact, some existing investigations into super recognition present evidence that supports this possibility, albeit with very small sample sizes (Bobak, Bennetts, et al., 2016; Bobak, Dowsett, & Bate, 2016; Bobak et al., 2016).

Such studies have assessed face perception skills in SRs using a variety of paradigms. For instance, the landmark SR paper of Russell et al. (2009) assessed face perception skills via the Cambridge Face Perception Test (CFPT; Duchaine et al., 2007). This test presents sets of six faces that have each been morphed to a different level of similarity from a target face. In each trial, participants are required to sort the faces in terms of their similarity to the identity of the target. While this test is frequently used to assess facial identity perception impairments in prosopagnosia (Bate & Tree, 2017; Bowles et al., 2009; Dalrymple & Palermo, 2016), it is not suitably calibrated for the detection of more able participants. Indeed, the large variability (and correspondingly large standard deviation) that has been observed in the performance of control participants prevents single-case comparisons at the top end from reaching significance (Bobak, Pampoulov, & Bate, 2016), and the reliability of the test has not yet been examined. Further, the very discrete artificially manipulated differences between images do not resemble a typical real-world face perception task, and the precise perceptual processes that are being assessed by the test remain unclear.

Other researchers have used face matching tasks to assess face perception, where participants are required to decide whether simultaneously presented pairs of faces display the same or different identities (e.g. Bobak, Dowsett, & Bate, 2016; Davis et al., 2016; Robertson et al., 2016). Another investigation assessed SRs on the well-used “One-in-Ten” test (Bruce et al., 1999), where participants are required to decide whether a target face is present within simultaneously presented line-ups containing 10 faces (Bobak et al., 2016). The studies reported by Bobak and Davis subsequently found that only some individuals outperformed controls on measures of face perception. Thus, because current protocols initially require superior performance on a test of face *memory* for experimental inclusion as a SR, the only available evidence suggests that superior face memory skills can present without superior face perception skills, and the converse has not yet been investigated. This clearly has both theoretical (e.g. in testing the assumptions of hierarchical accounts of face-processing) and practical (e.g. when seeking police officers who are proficient at particular face recognition tasks) importance; and further investigation into the patterns and prevalence of different subtypes of super recognition is sorely needed, using a wider variety of screening tests.

It is also pertinent that some inconsistencies have been observed in the performance of SRs across multiple measures of face memory or face perception (Bobak, Bennetts, et al., 2016; Bobak et al., 2016; Davis et al., 2016). This may indicate that some individuals achieve superior scores on a single attempt at a single test simply due to chance, and further testing reveals their true, more average abilities. Alternatively, differences in paradigm may bring about inconsistencies in performance, as has already been illustrated for face perception (i.e. in the use of the CFPT versus face matching tasks; see Bobak, Pampoulov, & Bate, 2016). That is, some individuals may have skills that are only suited to certain face-processing tasks, and this hypothesis may also extend to tests of face memory. For instance, all images of each individual identity in the CFMT+ were collected under tightly controlled conditions on the same day (Duchaine & Nakayama, 2006; Russell et al., 2009). Although some variability was incorporated into the greyscale images via changes in viewpoint, lighting, expression or the addition of noise, these manipulations do not capture the same variability that presents between images of the same person that have been collected on different days in a variety of naturalistic settings. Further, the CFMT+ only presents target-present trials, and does not assess the frequently encountered real-world scenario where a target face is actually absent. While another test used by Russell et al. (2009) may circumvent the former issue, it does not overcome the latter. Specifically, a “Before They Were Famous” test required participants to identify adult celebrities from childhood photographs, but no target-absent trials were included. Perhaps more fundamentally, the test is hampered by the difficulty of objective assessment across individuals due to potentially large differences in lifetime exposure to the target celebrities.

Finally, it could be argued that self-reported evidence of everyday face recognition may be used as a potential means to identify SRs. Such evidence could be collected anecdotally, or through more formal self-report questionnaires. Yet this issue of metacognition, particularly in relation to face recognition, has been much debated. While there is some evidence that self-report of everyday face recognition performance may be used as an approximate gauge of face recognition skills in the typical population (Bindemann, Attard, & Johnston, 2014; Bowles et al., 2009; Gray, Bird, & Cook, 2017; McGugin, Richler, Herzmann, Speegle, & Gauthier, 2012; Rotshtein, Geng, Driver, & Dolan, 2007) and those who may have prosopagnosia (e.g. Shah, Gaule, Sowden, Bird, & Cook, 2015), such investigations tend to only have mild-to-moderate effect sizes, and there is ample evidence and arguments to the contrary (e.g. Duchaine, 2008; Palermo et al., 2017; Tree, 2011). However, this issue has not yet been investigated at the top end of the face recognition spectrum, and it is possible that these individuals have a more accurate awareness of the level of their face recognition skills compared to those with typical or impaired abilities.

In sum, SRs need to be reliably identified for both theoretical and applied investigations, yet existing tests and protocols are open to criticism. As already stated, the main criterion for inclusion in a SR sample is superior performance on the CFMT+. While this procedure may overlook any candidate who is proficient only at face perception and not at face memory, it may also be overly simplistic by only taking one score on a single test at a single point in time as the critical measure. Indeed, some individuals may perform in the superior range on that occasion simply by chance, whereas others may fall short of the cut-off value due to extraneous variables such as fatigue, illness or simply “having a bad day”. Examining the consistency of performance across a variety of more applied tests that tap the same and different components of face-processing will address this issue, and ensure that the correct individuals are allocated to specific tasks in real-world settings.

The current paper set out to address these issues in a large number of adult Caucasian participants who had self-referred to our laboratory in the belief that they have superior face recognition skills. Because of the large sample size and diverse geography of the participants, the study was carried out online. In order to examine the accuracy of self-selection for SR research, we initially calculated the proportion of our sample who objectively met at least one criterion for super recognition. We then investigated the heterogeneity of super recognition by looking for dissociations between measures of face memory and face perception (although note that the consistency of face perception skills was not assessed across tests in the current paper). However, because our testing battery contained both traditional and more applied tests, we were able to examine consistency of performance across different measures of assessment.

## Methods

### Participants

Following large-scale media coverage of our previous work, a large number of individuals self-referred to our laboratory (via our website: www.prosopagnosiaresearch.org) in the belief that they possess superior face recognition skills. All participants were invited to take part in the screening programme, and 424 subsequently completed all four of the tests that are described in this paper. However, 224 participants were excluded from the final dataset to leave a sample size of 200 (140 female; age range 18–50 years; M = 37.2, SD = 7.7). Exclusions were made on the basis of age (> 50 years), ethnicity (only Caucasian participants were retained—if non-Caucasian participants were included in the study, renowned own-race biases in face recognition suggest that independent, appropriately matched control groups would be needed; e.g. Meissner & Brigham, 2001), reported assistance with the tests, self-reported or computer-reported technical problems, and previous exposure to the CFMT+. All participants took part in the study online and on a voluntary basis, motivated by the desire to discover whether they fit the criteria for super recognition. This group of individuals as a whole is referred to as the “experimental group” for the remainder of this paper.

Forty control participants (20 male) also participated in this study. Their mean age was 33.4 years (range 18–50 years, SD = 10.2), and these participants were compensated for their time in order to ensure their motivation on the tasks. Because it is possible that differences in performance may be noted between online and laboratory-tested participants, we tested half of these participants (10 female) online and the remaining half under laboratory conditions.

### Materials

Four objective tests were used in this investigation: the pre-existing CFMT+ and three new tests that were developed for the purposes of this study. The latter tests were designed to reflect more ecologically valid face recognition tasks, particularly those that may be encountered in policing scenarios. All tasks were designed to be carried out as accurately as possible, although, in an attempt to avoid particularly long response latencies, participants were informed that completion times would also be analysed. However, because the overall aim of this paper is to examine patterns of accuracy across tests, we only focus on this measure.

### The CFMT+ (Russell et al., 2009)

This test is an extended version of the original CFMT (Duchaine & Nakayama, 2006), a dominant test of unfamiliar face recognition that uses tightly controlled greyscale facial images. In the standard test, participants initially encode the faces of six unfamiliar males. Three views of each target face are shown (frontal, and left and right profiles) for 3 s each, and participants are immediately required to select the identical images from three triads of faces. Eighteen points are available for this section, and most typical participants receive full marks (Bobak, Pampoulov, & Bate, 2016; Bowles et al., 2009)—an unremarkable feat given that the task simply requires pictorial recognition following a minimal delay. Participants then review all six target faces again for a duration of 20 s. They are subsequently required to select a target face from 30 triads of faces, now presented from novel viewpoints or lighting conditions. After another 20-s review of the target faces, 24 further triads are presented, with noise overlaid onto the images. The CFMT+ extends this section by including an additional 30 triads with more extreme changes in facial expression or viewpoint, providing a total score out of 102. All triads in the test contain a target face, and some distractors are repeated to enhance difficulty. Participants make responses using the 1–3 number keys on a keyboard, and triads remain on-screen until a response is made. Reaction time is not monitored.

### Models memory test (MMT)

**Fig. 1:**
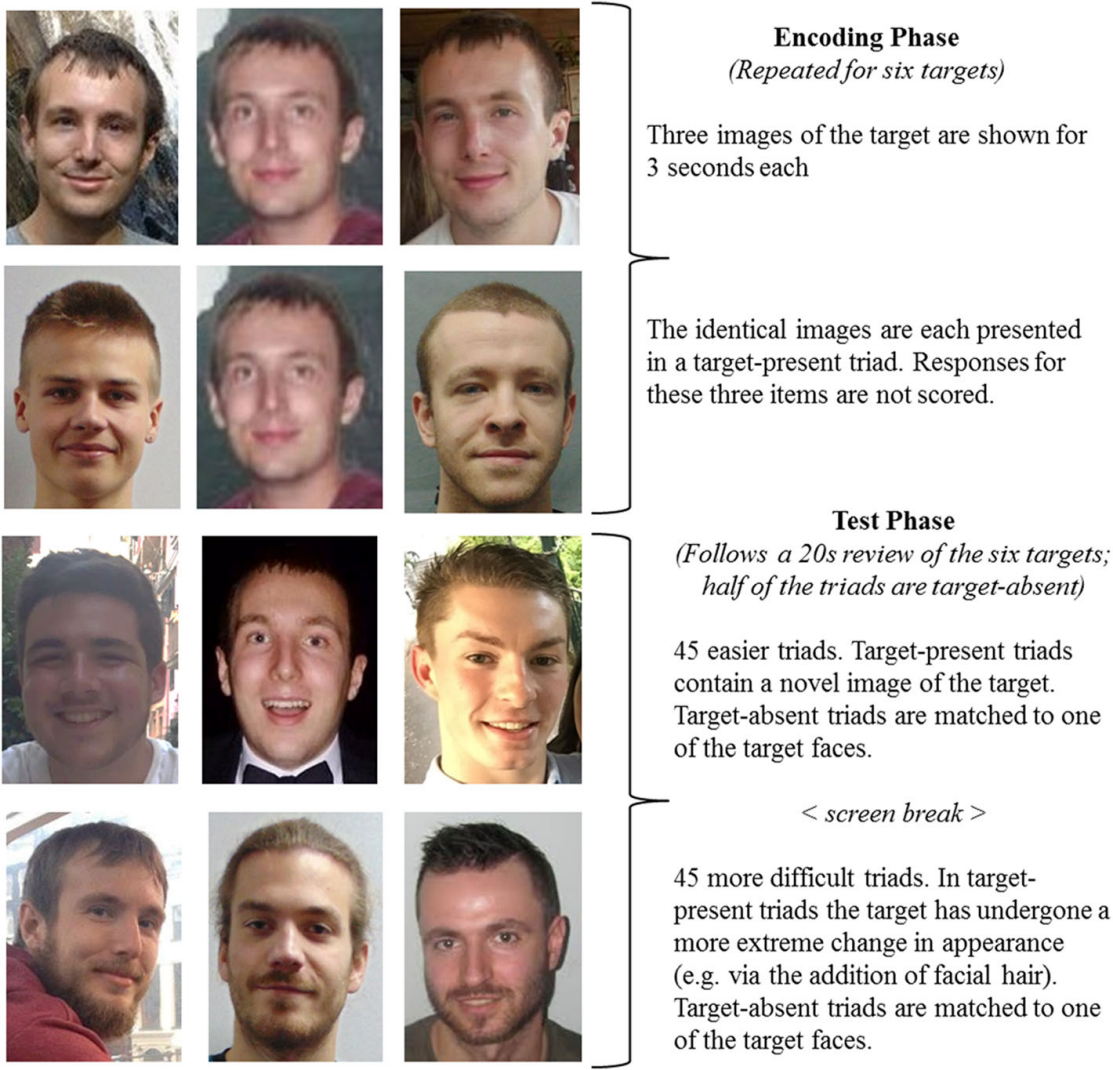
Sample stimuli from the MMT. Note that these trials are all target-present. Due to issues with image permissions, this figure only displays images that resemble those used in the actual test

This new test of face memory was developed in our laboratory for the purposes of this study. While the CFMT+ uses tightly controlled facial images, our new test was designed to embrace the more real-world natural variability that occurs between different presentations of the same face (Young & Burton, 2018, 2017). We therefore used a variety of more naturalistic, colour images of each person, taken on different days and in very different scenarios. To collect these images, we adopted the procedure used by Dowsett and Burton (2015) to acquire 14 very different facial images of each of six young adult males, via the webpages of modelling agencies (see Fig. 1). We used the same technique to collect a pool of 300 unique distractor faces, which were combined with the target images to create the testing triads (see later). We used faces that were all of the same gender to maintain difficulty across trials (i.e. we did not want to half the number of candidate faces, or even double the number of stimuli, by including both genders). Because gender biases have only been shown for the recognition of female and not male faces in previous work (e.g. Herlitz & Lovén, 2013; Lovén, Herlitz, & Rehnman, 2011), we followed the precedent of the CFMT+ by only using male faces. All images were cropped from just below the chin to display the full face, and, to mimic real-world face recognition, none of the external features was excluded. Each image was adjusted to dimensions of 8 cm in height and 6 cm in width.

Our new test maintained a similar encoding procedure to that used in the CFMT+ (see Fig. 1): for each target face, three different images are each presented for 3 s, followed by three test triads where participants are required to select the repeated image. However, instead of each face being initially shown from three viewpoints, we displayed three frontal images of each face that were taken on different days in very different settings. To create the testing triads, each image of a target was matched to two distractor faces from the pool, according to their external facial features and viewpoint. These 18 encoding trials do not contribute to the overall score. We did maintain the first 20-s review of the six target faces from the CFMT+ (presented immediately after the encoding phase), but displayed a new frontal image of each target that was again taken on a different day and in a different environment.

Participants then received 90 test trials (45 target-present), in a random order for each participant, with a screen break after the first 45 trials. The inclusion of target-absent trials differs from the CFMT+. Because the latter is a direct development of a test that is designed to detect prosopagnosia, the inclusion of target-absent trials may result in low-ability participants eliciting this response on every trial. However, those who are truly at the higher end of the spectrum should be adept at both correct identifications and correct rejections—as is required in policing scenarios and in real-life interactions. Thus, by including both target-present and target-absent trials, our new test provides a more encompassing assessment of participants’ face recognition abilities. As in the encoding phase, very different images of each target were included in the test triads. We collected a further five to seven images of each target face, and five distractor faces were selected from the pool that were considered to match each individual target image. Two were combined with the target image to form a target-present triad, and the remaining three were combined to form a matched target-absent triad. The resulting 90 triads were then divided into two equal groups, with the first containing images that were more similar to the encoding images of the target faces (i.e. those taken from similar viewpoints with minimal changes in facial appearance) and the second containing triads with more abrupt changes (i.e. the addition of facial hair or accessories that obscured part of the face, or a more dramatic change in viewpoint).

For each trial, participants were required to respond with the corresponding number key (1–3) to indicate the position of a target in the triad, or with the 0 key if they believed the triad to be target-absent. As in the CFMT+, each triad remains on-screen until a response is made. After completing the first 45 trials, participants view an instruction screen that invites them to have a brief rest before beginning the final, more challenging phase of the test. However, contrary to the CFMT+, this phase does not commence with an additional review of the target faces, in order to maintain the enhanced difficulty of the task.

Because of the inclusion of target-absent trials, five different categories of responses are possible in the task. In trials with a target face present, participants’ responses can be categorised as either hits (correctly identifying the target face), misses (incorrectly saying that a target face was not present) or misidentifications (incorrectly identifying one of the distractor faces as a target). In trials without a target face, responses can be categorised as either correct rejections (correctly stating that no target face was present) or false positives (incorrectly identifying one of the faces as a target). Each of these measures was calculated separately for each participant, along with an overall accuracy score (the sum of hits and correct rejections).

### Pairs matching test (PMT)

**Fig. 2:**
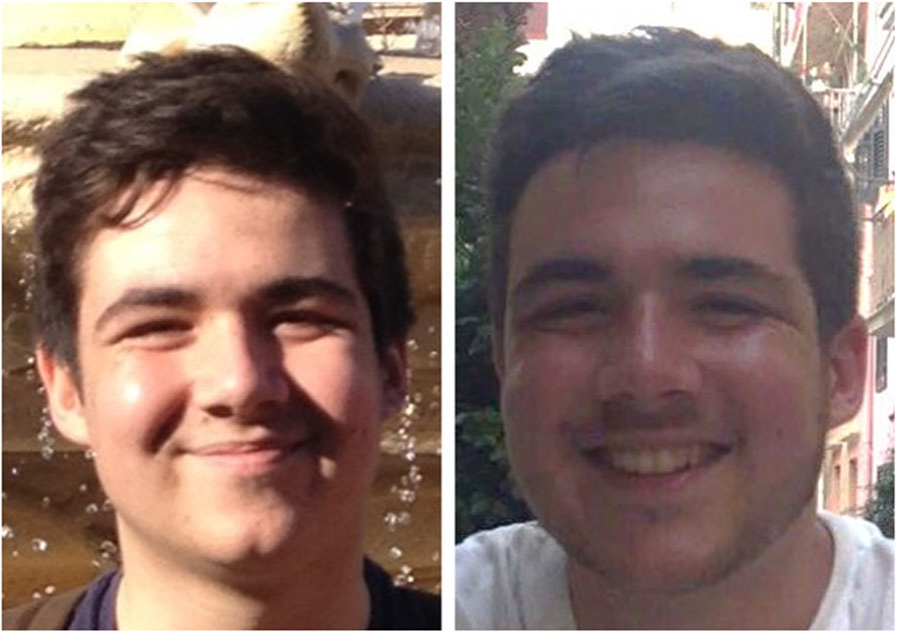
A sample pair from the PMT. The two identities differ in this trial. Due to issues with image permissions, this figure only displays images that resemble those used in the actual test

This test was created in our laboratory using a very similar design to existing face matching tests (e.g. Burton, White, & McNeil, 2010; Dowsett & Burton, 2015), but with enhanced difficulty. The creation of a new, sufficiently calibrated test was necessary so that we could confidently detect top performers via single-case statistical comparisons. We created 48 colour pairs of faces (24 male), half of which were matched in identity (see Fig. 2). As in the previous test, all images were downloaded from the websites of modelling agencies. To ensure difficulty of the test, the faces in the mismatched trials were paired according to their perceived resemblance to each other. All images were cropped to display the full face from just below the chin, and all external features were included. Images were adjusted to 10 cm in width and 14 cm in height. The test displayed each pair of faces simultaneously, and participants were required to make a key press indicating whether the faces were of the same individual or two different individuals. To replicate the demands of this task in everyday and occupational settings (e.g. passport control, CCTV image matching) no time limit was imposed in making a response, and the pair stayed on the screen until a response was made. For each participant, trials were randomised and presented within a single block.

### Crowds matching test

**Fig. 3:**
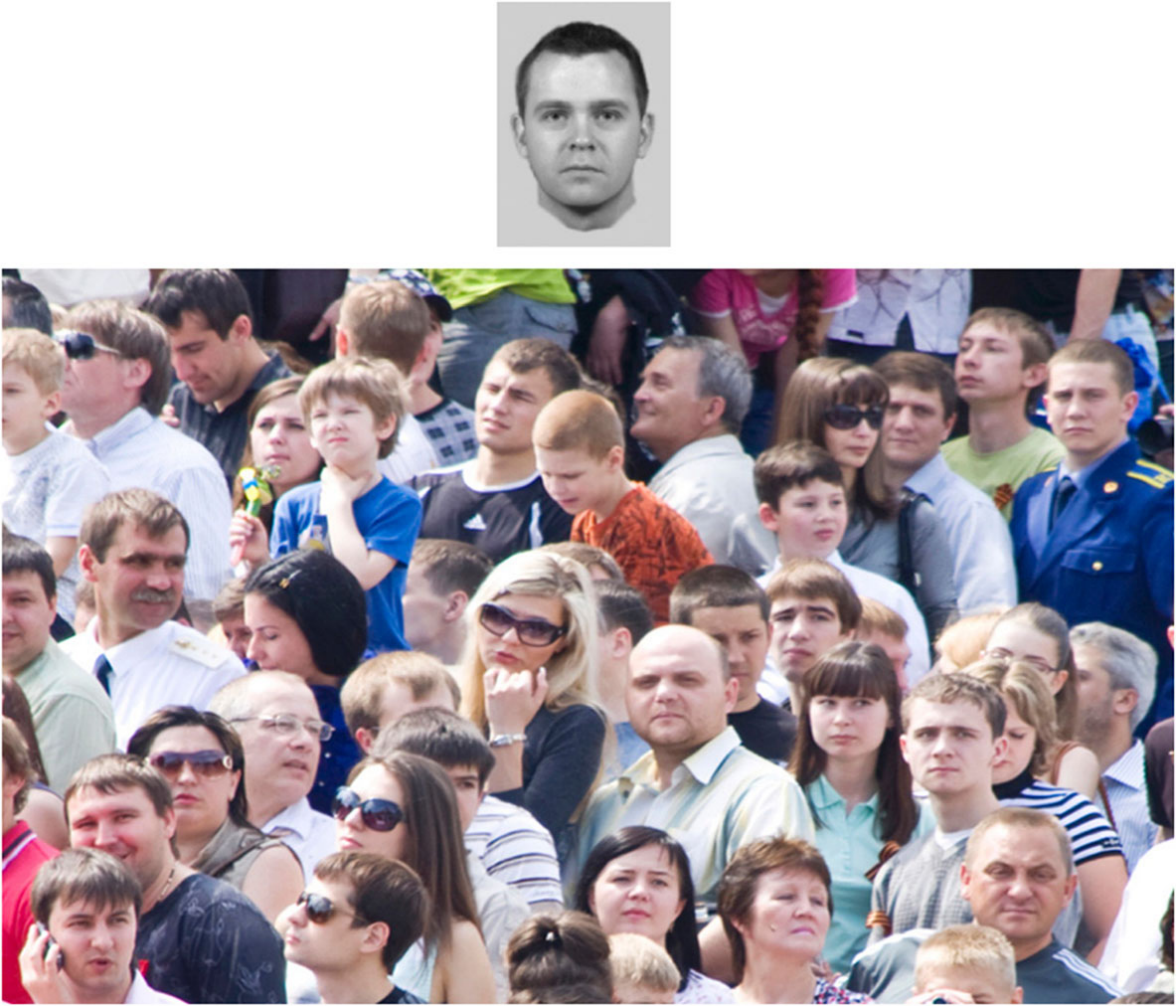
A sample target-present trial from the Crowds test

We developed a new test of face matching that required participants to decide whether a composite target face is present within a simultaneously presented image displaying a crowd of people. The crowd images displayed 25–40 people in a variety of scenarios, such as watching sports matches or concerts, or running in a marathon (see Fig. 3). The test was designed to simulate a policing scenario where officers or police staff might have a composite image of a perpetrator and are searching for him or her in a crowd or within CCTV footage. Thirty-two trials (16 target-present) were presented in a random order, with a single composite (measuring 3 cm in height and 2 cm in width) displayed at the top of the screen and a crowd image (measuring 9 cm in height and 13 cm in width) beneath (see Fig. 3). Participants had an unlimited time to decide, via a single keyboard response, whether the identity depicted by the composite was present in the crowd scene.

We made use of the EvoFIT holistic system, in current police use, as the resulting faces can be readily named by other people (e.g. M = 45% correct in Frowd et al., 2012). Constructors repeatedly select from arrays of alternatives, with choices combined, to allow a composite face to be “evolved”; the procedure involves focus on the internal features of the face, the area that is important for familiar-face recognition (e.g. Ellis, Shepherd, & Davies, 1979) and naming of the composite. We used a standard face-construction protocol (Frowd et al., 2012), as undertaken by real witnesses and victims of crime, and the composites were constructed by different participants after each person had seen an unfamiliar target face. As the procedure for set up of the stimuli (including composite face construction) is fairly involved, full details are provided in Additional file 1.

### Procedure

The experimental group initially filled in an online questionnaire that enquired about background demographical information and checked each participant’s belief that they have superior face recognition skills. They were then sent online links to the four objective tests, which they completed in a counterbalanced order. After all tests were complete, participants were sent a “quality control” questionnaire that asked whether they had experienced any technical problems during completion of the tests, if they had received any assistance from other people and whether they have previously completed the CFMT+.

Control participants were recruited via Bournemouth University’s established participant network, and were randomly allocated to either the online or laboratory condition. Those who completed the tests online were sent the links to the tests in the same manner as the experimental group. Laboratory participants completed all tests on the same online platform, but under monitored experimental conditions.

### Statistical analyses

Initial analyses were carried out on the performance of the control participants to detect whether there was any differences in performance between online and laboratory participants. Akin to previous work (e.g. Germine et al., 2012), no differences were detected on any test (all *p*s > .55) and control data were subsequently collapsed across the two groups of participants for comparison to the experimental group. As there were no significant differences in age between the two control groups, or in comparison to the experimental group, we did not further sub-divide the participants according to age. Indeed, existing work indicates consistency in adult performance until the age of 50 (e.g. Bowles et al., 2009), the upper age limit for all of our participants.

Performance on each of the four tests was initially calculated in terms of overall accuracy. Because the three new tests (i.e. all but the CFMT+) contained target-present and target-absent trials, these items were also analysed separately, together with relevant signal detection measures (see later). Mean and SD scores were calculated for all performance measures, and cut-off values were set at ± 1.96 SDs from the control mean (see Table 1). In the following, the term “SR” is used to refer to individuals from the experimental group who surpassed the relevant cut-off value.

**Table 1:** Control norms (*N* = 40) for overall performance on each test

	Maximum score	Chance	Control mean (SD)		Cut-off value	
			Proportion correct	Raw score	Proportion correct	Raw score^a^
CFMT+	102	.33	.68 (.10)	69.53 (10.02)	.87	90
MMT	90	.25	.54 (.14)	48.43 (12.44)	.81	73
PMT	48	.50	.69 (.07)	33.03 (3.49)	.83	40
Crowds test	32	.50	.63 (.12)	20.13 (3.76)	.86	28

*CFMT+* long form of the Cambridge Face Memory Test, *MMT* models memory test, *PMT* pairs matching test, *SD* standard deviation

^a^Raw score cut-off values rounded up to the next whole number that is 1.96 SDs from the control mean. This score is taken as the cut-off value to determine superior performance. Note that cut-off values were calculated prior to rounding

## Results

### Performance on the CFMT+

**Fig. 4:**
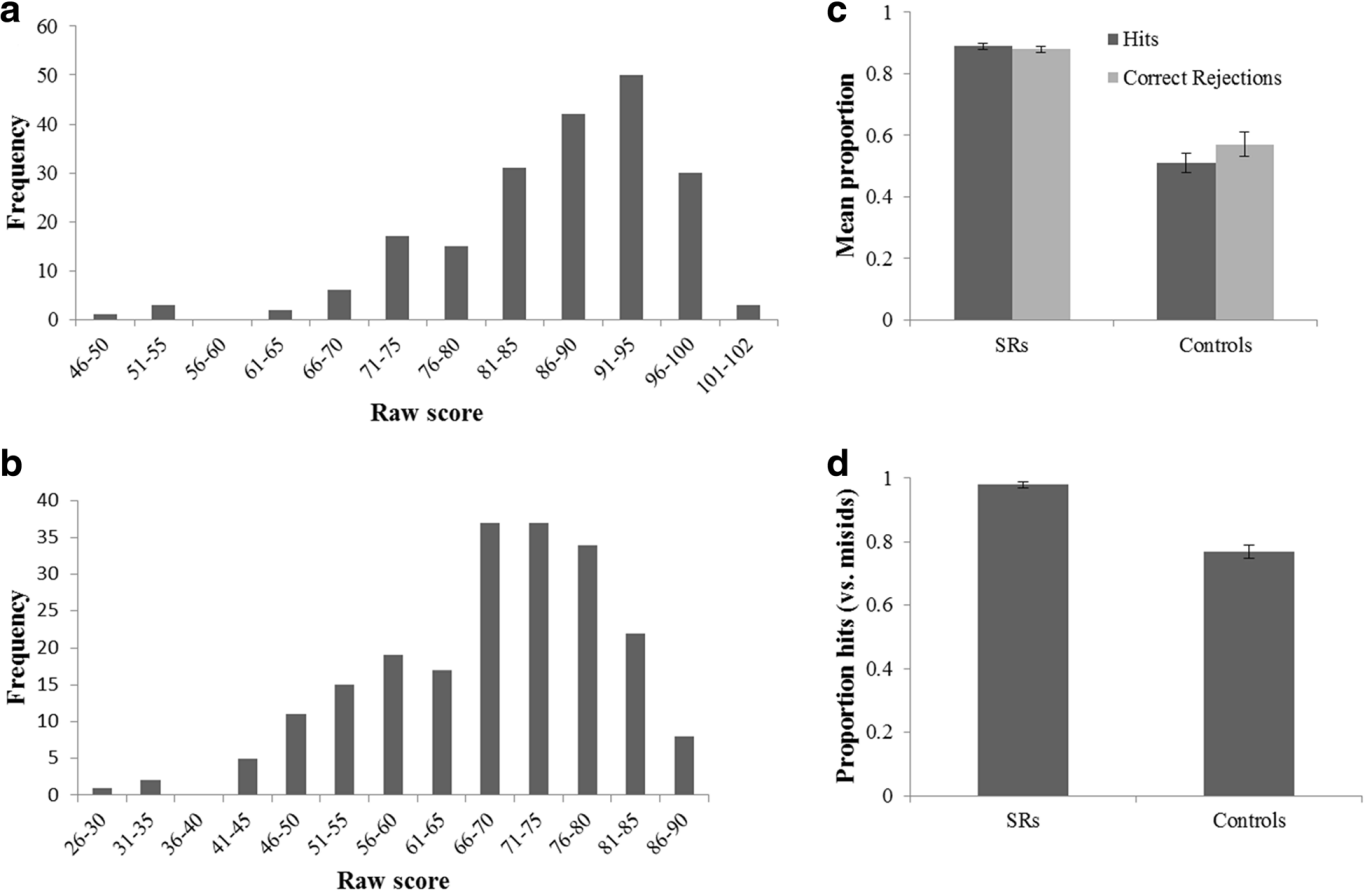
Performance on the face memory tests. Distribution of experimental group’s performance on the (**a**) CFMT+ and (**b**) MMT, and the proportion and standard error of hits (**c**), correct rejections (**c**) and positive responses in target-present trials that were hits (vs misidentifications) (**d**) made by “super recognisers” (SRs) in comparison to controls on the MMT

Performance of our control group on the CFMT+ yielded norms (see Table 1) that are a little lower than those generated by previous work (e.g. a raw score cut-off value of 95 was presented by Bobak, Pampoulov, & Bate, 2016—this figure was calculated following laboratory testing of 254 young adults). The 200 participants in the experimental group scored in the range of 50–102 (out of a maximum score of 102; see Fig. 4a), with 89 individuals (44.50%) exceeding the criterion for superior performance.

### Performance on the MMT

Because the three new tasks contained both target-present and target-absent trials (or match/mis-match for the PMT), the analysis proceeded in two steps (see Table 2). First, we examined the overall accuracy of the experimental group individually, to identify high-performing individuals who excelled at the specific task. For the MMT, the experimental group performed in the range of 27–90 (maximum score was 90), with 85 participants achieving superior performance (henceforth SRs; see Fig. 4b).

**Table 2:** Breakdown of performance on the models memory test

	Control mean (SD)	SR mean (SD)
Target-present trials: proportion of hits	.51 (.20)	.88 (.07)
Target-absent trials: proportion of correct rejections	.57 (.23)	.88 (.08)
Target-absent trials: proportion of misidentifications	.15 (.11)	.02 (.02)
Target-absent trials: proportion of misses	.34 (.21)	.10 (.07)
Overall proportion correct	.54 (.14)	.88 (.05)
*d′* (sensitivity)	0.26 (0.84)	2.46 (0.59)
*c* (bias)	−0.12 (0.61)	−0.06 (0.33)
Proportion of positive responses in TP trials that were hits (vs misidentifications)	.77 (.15)	.98 (.02)

*SD* standard deviation, *SR* “super recogniser”

Second, we conducted signal detection-based analyses to compare performance between the group of 85 individuals who demonstrated superior performance on this test and the control group. To do this, we generated scores of sensitivity (*d′*) and bias (*c*) for each participant. The measure *d′* incorporates information from hits and false positives to create a measure of sensitivity that is free from the influence of response bias (Macmillan & Creelman, 2005). A score of 0 indicates chance performance, and values for the current test can range from − 4.59 (consistently incorrect responding) to + 4.59 (perfect accuracy). The measure *c* is used as an indicator of response bias (i.e. whether the participant has a tendency to say that the target is present or absent; MacMillan & Creelman, 2005). A score of 0 indicates a neutral response criterion, whereas a positive score indicates conservative responding (a tendency to indicate that a target was not present) and a negative score indicates more liberal responding (a tendency to indicate that a target was present). For this analysis, we incorporated all instances when the participant indicated that a target was present, even when their identification of the target was incorrect (i.e. we included both hits and misidentifications for target-present trials, to calculate a measure of response bias that indexed a tendency to indicate that a target was present/absent overall). Scores for *d′* and *c* were corrected using the loglinear approach proposed by Stanislaw and Todorov (1999).

There was a significant difference between the high-performing group and controls for *d′*, *t*(123) = 16.875, *p* = .001, *d* = 3.03, but not bias, *t*(123) = 0.722, *p* = .471. Follow-up analyses were carried out to analyse the pattern of responding in more detail. A two-way mixed ANOVA with group (SRs and controls) and correct response type (hits and correct rejections) confirmed that, averaged across the two types of responses, SRs outperformed controls, *F*(1,123) = 408.012, *p* = .001, ηρ^2^ = .768, but there was no main effect of response type nor a significant interaction between group and the type of correct response, *F*(1,123) = 1.320, *p* = .253 and *F*(1,123) = 2.563, *p* = .112, respectively (see Fig. 4c). In other words, the effects were not driven disproportionately by correct responses on target-present or target-absent trials. Furthermore, the SRs made proportionately fewer misidentification errors than the control group, *t*(1,123) = 9.925, *p* = .001, *d* = 1.54 (see Fig. 4d). This pattern held when analysing the raw number of misidentifications, and also when the number of misidentifications was controlled for by the number of overall positive identifications in target-present trials (by calculating the proportion of positive responses in target-present trials that were hits vs misidentifications), *t*(123) = 12.220, *p* = .001, *d* = 3.03.

Overall, this pattern of responses suggests that the participants who performed well on the MMT did so because they were capable of identifying the target faces more accurately when they were present, and correctly identifying when they were absent; this outcome is as opposed to either showing a general response bias or a tendency to indicate that a target face was present (regardless of whether they could subsequently identify the familiar face).

### Performance on the PMT

**Fig. 5:**
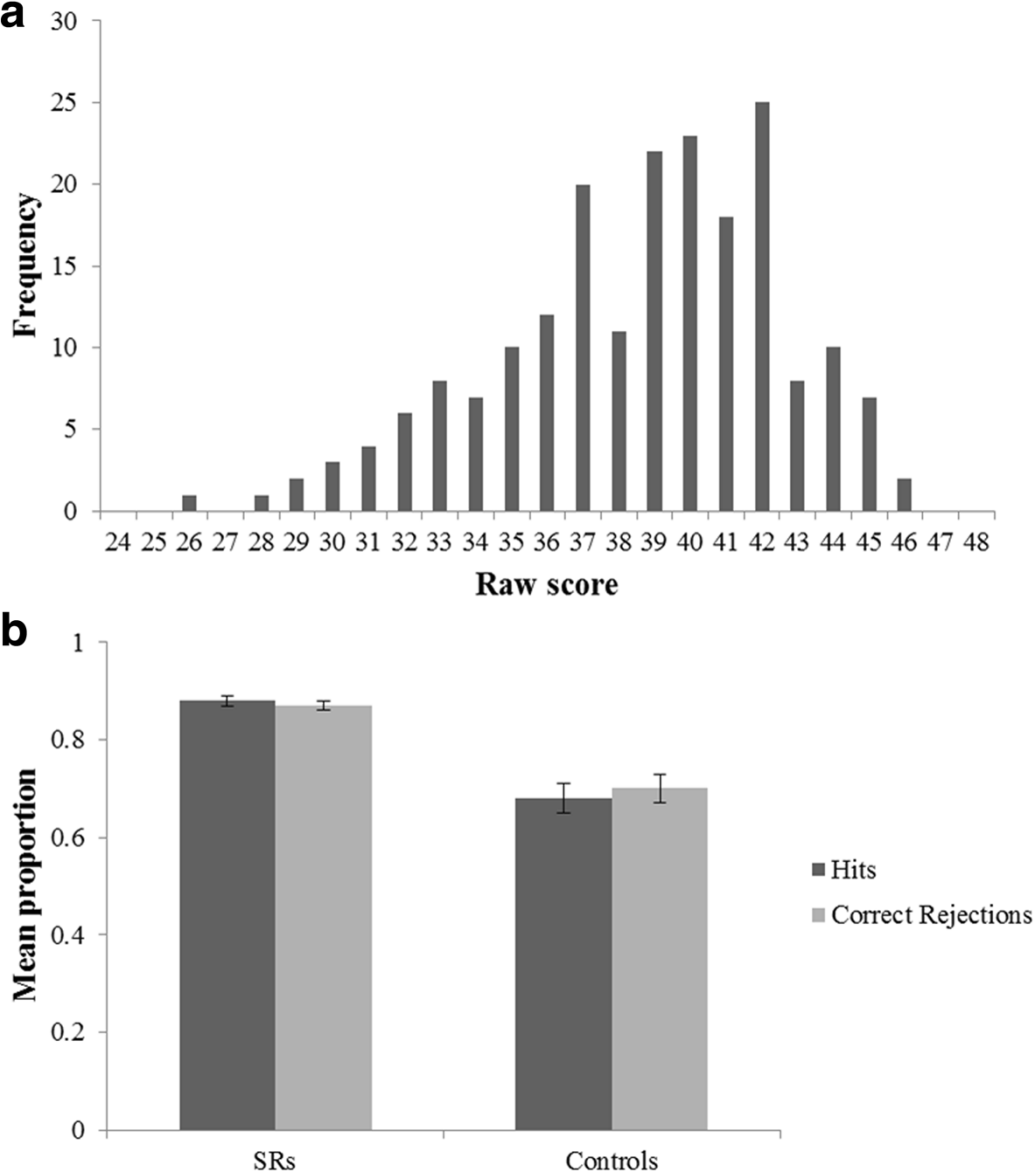
Performance on the PMT. Distribution of experimental group’s performance on the PMT (**a**), and number of hits and correct rejections made by “super recognisers” (SRs) in comparison to controls (**b**)

The experimental group’s performance on the PMT ranged from 26 to 46 correct out of a possible 48 (see Fig. 5a). Ninety-three participants exceeded the criterion for superior performance on this test. As in the MMT, we calculated accuracy separately for the different trial types (hits, correct responses in “same” trials; false positives, incorrect responses in “different trials”) and used these to calculate SDT measures (see Table 3). Due to the clearly non-normal distribution (negative skew) of the data, the analysis for this task used alternative, non-parametric measures of sensitivity (*A*) and bias (*b*) (Zhang & Mueller, 2005). The measure *A* ranges from 0 (chance performance) to 1 (perfect performance); values of *b* (positive vs negative scores) are interpreted similarly to criterion *c*.

**Table 3:** Breakdown of performance on the pairs matching test

	Control mean (SD)	SR mean (SD)
Proportion of hits	.68 (.17)	.88 (.08)
Proportion of correct rejections	.70 (.16)	.87 (.08)
Overall proportion correct	.69 (.07)	.87 (.03)
*A* (sensitivity)	.73 (.08)	.85 (.08)
*b* (bias)	1.09 (.45)	1.10 (.42)

*SD* standard deviation, *SR* “super recogniser”

Similarly to the MMT, the analysis of sensitivity (*A*) was significant, *t*(131) = 7.715, *p* = .001, *d* = 1.50, whereas the analysis of bias (*b*) was not, *t*(131) = 0.114, *p* = .909. SRs showed significantly better performance than controls, but there was no difference between the groups in response bias. Once again, we conducted follow-up analyses on the proportion of hits and correct rejections for each group using a two-way mixed ANOVA. While there was a significant main effect of group, *F*(1,131) = 392.472, *p* = .001, ηρ^2^ = .750, there was no main effect of response type nor significant interaction between the two, *F*(1,131) = 0.122, *p* = .727, and *F*(1,131) = 0.309, *p* = .579, respectively. This finding indicates that there was no significant difference in the proportion of hits versus correct rejections for these individuals compared to controls (see Fig. 5b).

### Performance on the Crowds test

**Fig. 6:**
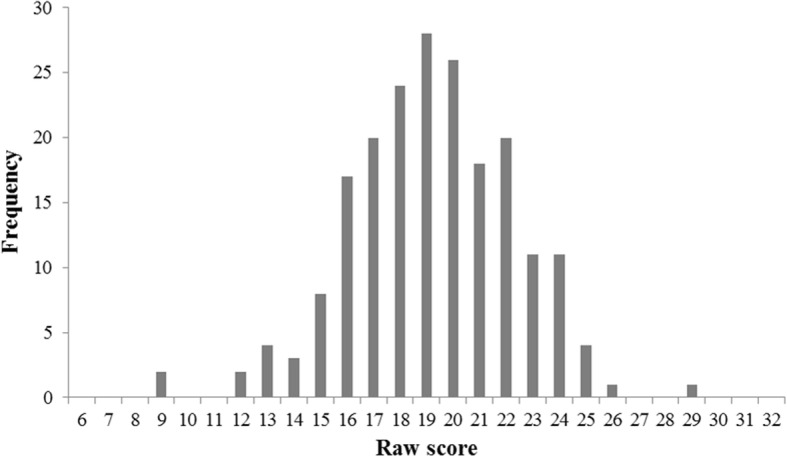
Distribution of experimental group’s performance on the Crowds test

The experimental group’s performance on the Crowds test was much more varied, with overall accuracy scores ranging from 9 to 29 out of a possible 32 (see Fig. 6); these data indicate performance which appeared to align nicely with a normal distribution with little skew. Only one participant outperformed controls on this task (see Table 4). Examination of the different types of responses in more detail revealed that controls made a similar number of hits (M = 9.73, SD = 2.57) compared to correct rejections (M = 10.40, SD = 2.79), *t*(39) = 1.101, *p* = .278; and the one superior performer achieved 14 and 15, respectively. No control participant exceeded the cut-off value of 1.96 SDs, but the second top-performing control was 3 points short of this cut-off, and was the only control participant to reach the superior range on the CFMT+. This individual also performed at 1.5 SDs above the control mean on the MMT, but performed very closely to the control mean on the PMT.

**Table 4:** Control mean (SD) and SR (*N* = 1, only one individual outperformed controls on this task) scores on the Crowds test

	Control mean (SD)	SR score
Proportion of hits	.61 (.16)	.88
Proportion of correct rejections	.65 (.17)	.94
Overall proportion correct	.63 (.12)	.91
*d′* (sensitivity)	0.68 (0.62)	2.40
*c* (bias)	0.06 (0.34)	0.15

*SD* standard deviation, *SR* “super recogniser”

These results suggest that it is difficult to surpass the control cut-off value on the Crowds task. Indeed, as argued in the Discussion, composites constructed from memory (as is the case here) are usually difficult to recognise or match to target. Given that 1.96 SDs from the control mean may be simply too conservative a cut-off value on this test, we also examined the performance of individuals who performed more than 1 SD above the control mean. Seventeen members (8.5%) of the experimental group exceeded this cut-off value, as did a somewhat larger proportion of the control group (20%). Seven of the 17 experimental group members (41.1%) displayed superior performance on the PMT, while three others (17.6%) achieved a superior score on the CFMT+. If we consider the other end of the spectrum on the Crowds task, specifically for the lowest 17 performers, a very similar pattern occurred: six individuals achieved a superior score on the PMT, and three others on the CFMT+.

### Relationship between tests

The CFMT+ is a strictly controlled laboratory test of face recognition, and is the dominant means of detecting super recognition. Conversely, the three new tests were designed to reflect more applied face recognition tasks that are encountered in policing scenarios, and included target-present and target-absent trials. Our next set of analyses investigated the relatedness of the four tests, examining just the data from the experimental group, and then the entire dataset (i.e. including both experimental and control participants). First, scores for the experimental group were factor analysed using principal component analysis (PCA) with varimax (orthogonal) rotation. Because we were particularly interested in the value of target-absent trials in identifying SRs, we entered hits and correct rejections separately into the analysis for the three new tests. The overall score correct (i.e. hits) was entered for the CMFT+. The analysis yielded four factors that explained a total of 80.59% of the variance for the entire set of variables. Factor 1 had high loadings from the CFMT+ and hits from the MMT, and explained 29.49% of the variance (see Table 5). The second factor was derived from the hits and correct rejections on the Crowds test, and explained a further 27.29% of the variance. The third factor was only derived from the hits on the PMT, explaining 12.49% of the variance; and the fourth factor was only derived from the correction rejections on the MMT, explaining 11.33% of the remaining variance. A full correlation matrix is displayed in Table 6, further demonstrating the strong relationship between the CFMT+ and hits on the MMT, and mild associations between some of the other measures.

**Table 5:** Orthogonally rotated component loadings for factor analysis of the experimental group’s performance on the four face recognition tests, including hits and correct rejections

Component	1	2	3	4
CFMT+	0.91			
MMT: hits	0.88			
MMT: CRs				0.97
PMT: hits			0.91	
PMT: CRs				
Crowds: hits	−1.02	−.94		
Crowds: CRs	−1.07	−.64	0.49	

*CFMT+* long form of the Cambridge Face Memory Test, *CR* correct rejection, *MMT* models memory test, *PMT* pairs matching test

**Table 6:** Correlation matrix for the experimental group’s performance on the four face recognition tests, including hits and correct rejections

		CFMT+	MMT		PMT		Crowds test	
			Hits	CRs	Hits	CRs	Hits	CRs
CFMT+		1	.65*	.16	.20*	.25*	−.05	−.06
MMT	Hits		1	.03	.31*	.19	−.12	.06
	CRs			1	−.03	.31*	.14	−.25*
PMT	Hits				1	−.23*	−.25*	.35*
	CRs					1	.19*	−.36*
Crowds	Hits						1	−.44*
	CRs							1

*CFMT+* long form of the Cambridge Face Memory Test, *CR* correct rejection, *MMT* models memory test, *PMT* pairs matching test

*p < .008; Bonferroni correction applied

To further identify related factors underlying the battery of tests, we performed a PCA on the data collected from all participants (i.e. the entire experimental sample and the controls). Initial eigenvalues indicated that the first two factors explained 32.11% and 27.65% of the variance, and the remaining five factors had eigenvalues that were less than 1. Solutions for two, three and four factors were each examined using varimax and oblimin rotations of the factor loading matrix. The three-factor varimax solution (which explained 71.63% of the variance) was preferred, as it offered the best defined factor structure (see Table 7). Similarly to our initial factor analysis, the CFMT+ and hits from the MMT loaded heavily on the first factor. However, hits from the PMT also loaded heavily on this factor, suggesting it represents performance on target-present trials. The second factor has high loadings from performance on target-absent trials (i.e. correct rejections) in both the MMT and PMT. The final factor has high loadings from the Crowds test. Thus, this analysis more clearly differentiates between (a) target-present and target-absent performance on the CFMT, MMT and PMT, and (b) the Crowds test in relation to the other three tests.

**Table 7:** Factor loadings for combined performance of the control and experimental groups, based on principal components analysis with oblimin rotation

Component	1	2	3
CFMT+	.83	.35	
MMT: hits	.89		
MMT: CRs		.81	
PMT: hits	.65	−.31	−.33
PMT: CRs		.75	
Crowds: hits			.89
Crowds: CRs		−.38	−.68

Hits and correct rejections (CRs) entered separately where relevant

*CFMT+* long form of the Cambridge Face Memory Test, *MMT* models memory test, *PMT* pairs matching test

### Overall indices of performance

**Fig. 7:**
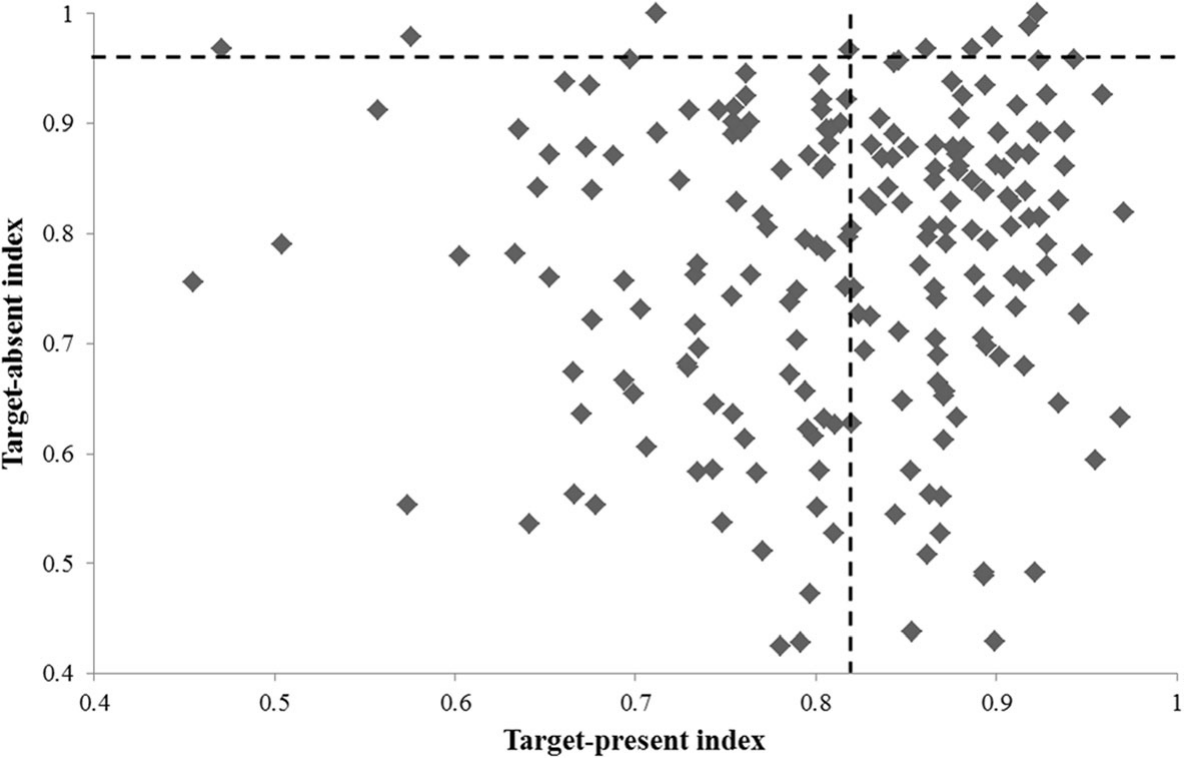
Correlation between the experimental group’s target-present and target-absent indices of performance

Because performance on target-present and target-absent trials loaded separately across the CFMT+, MMT and PMT, we created indices of target-present (by averaging the proportion of hits on the CFMT+, MMT and PMT) and target-absent (by averaging the proportion of correct rejections on the MMT and PMT) performance (see Table 8). Unsurprisingly, no significant correlation was observed between the two indices in either the experimental group or controls (*r* = .067, *p* = .346, and *r* = .109, *p* = .503, respectively) (see Fig. 7). Nine participants surpassed controls on the target-absent index, and 103 on the target-present index. Only five of these individuals exceeded the control cut-off value on both indices.

**Table 8:** Norming data from control sample for target-present and target-absent indices

	Mean	Standard deviation	Cut-off value
Target-present	.66	.08	.82
Target-absent	.63	.16	.96

**Fig. 8:**
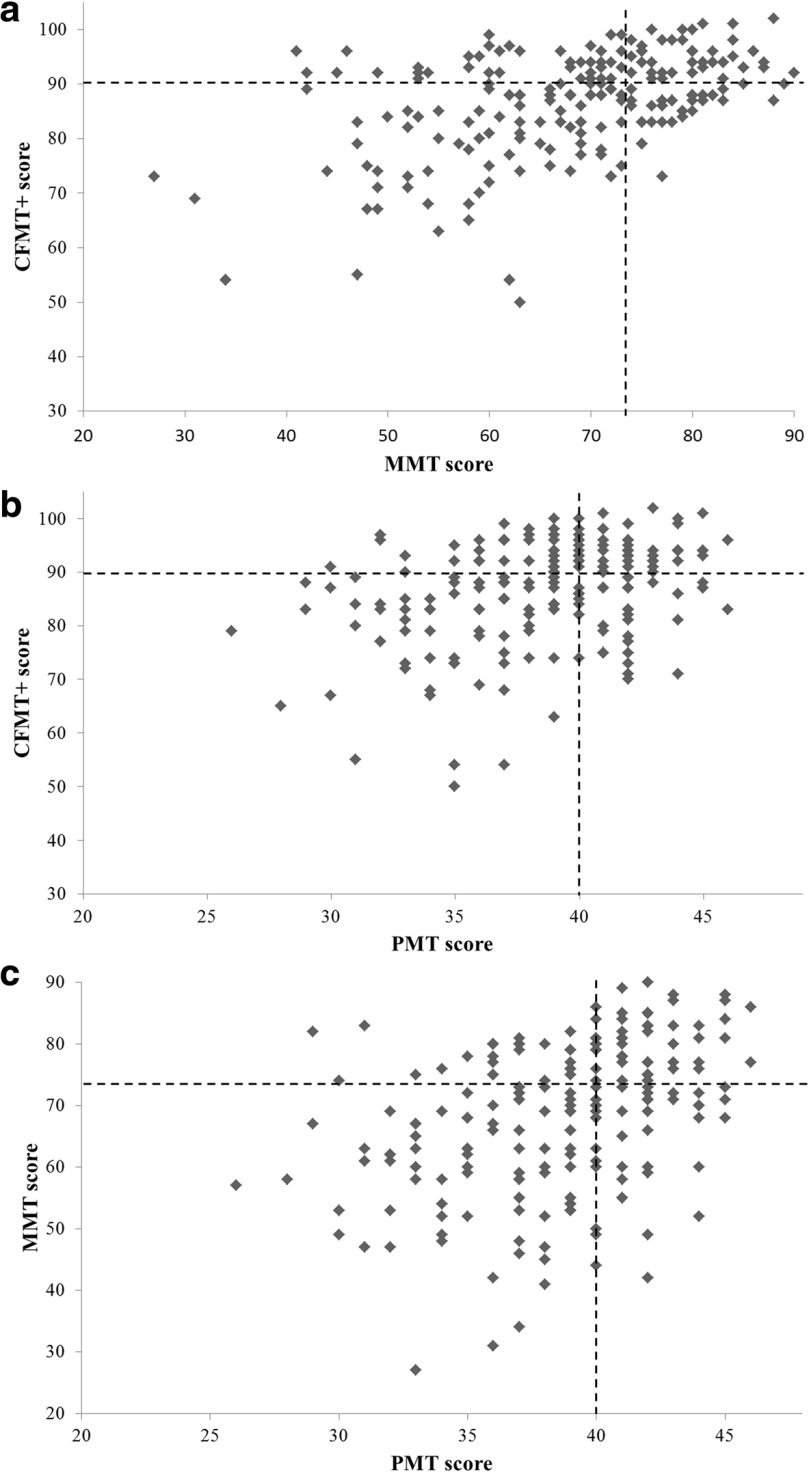
Correlations in performance. Correlation between the experimental group’s performance on the (**a**) CFMT+ and MMT, (**b**) CFMT+ and PMT, and (**c**) MMT and PMT. Dashed lines represent cut-off values for superior performance on each test. CFMT+ long form of the Cambridge Face Memory Test, MMT models memory test, PMT pairs matching test

Finally, we examined the consistency of superior performance across the tasks with regard to face memory versus face matching performance. In terms of face memory, a small but significant correlation was observed between performance on the CFMT+ and the MMT (*r* = .146, *p* = .039), and 49 participants scored within the superior range on both tests (see Fig. 8a). Larger correlations in performance were noted between the PMT and both the CFMT+ (*r* = .476, *p* = .001) and the MMT (*r* = .394, *p* = .001). Out of the 93 participants who significantly outperformed controls in the PMT, 74 also performed in the superior range on either the CFMT+ (*N* = 19; see Fig. 8b), the MMT (*N* = 18; see Fig. 8c) or both memory tests (*N* = 37; see Fig. 8b, c). Notably, however, 18 participants did not achieve superior scores on either memory test. To investigate whether a dissociation could be confirmed between face memory and face matching in these individuals, we used Crawford and Garthwaite’s (2002) Bayesian Standardized Difference Test to investigate whether, for each person, the difference between scores on the CFMT+ and the PMT was significantly larger than the mean difference between scores observed in controls. A significant difference between performances on the two tasks was noted in three participants (see Table 9). No significant differences were noted for the converse dissociation (i.e. in those who achieved superior scores on the CFMT+ and MMT but not the PMT) in the 13 individuals who displayed this pattern of performance. Finally, it is of note that the individual who excelled in the Crowds test also achieved a superior score on the PMT (42/48). However, the performance of this participant was very close to the control mean scores on both the CFMT+ (71/102) and the MMT (49/102).

**Table 9:** Dissociation between face matching and face memory performance in three “super matchers”

	Test scores			Bayesian Standardized Difference Test: CFMT+ vs PMT		
	CFMT+	MMT	PMT	*t*	p*	% population more extreme
SM1	.69	.58	.88	2.348	.024	1.20
SM2	.70	.67	.88	2.258	.030	1.48
SM3	.70	.76	.92	2.770	.009	0.43

*CFMT+* long form of the Cambridge Face Memory Test, *MMT* models memory test, *PMT* pairs matching test, *SM* “super matcher”

*Holm’s sequential Bonferroni correction applied

## Discussion

This study aimed to examine the consistency of performance of 200 self-referred SRs (the experimental group) across four face-processing tests. First, participants completed the dominant test of face memory that is currently used to identify SRs (the CFMT+). They then participated in three new more applied tasks that were designed to mimic face recognition scenarios that are encountered by the police: a test of face memory (the MMT), a face matching task (PMT) and a test that requires participants to spot a composite target face in a crowd (Crowds test). When results from each test were examined independently, 37 people achieved consistently superior scores across three of these tests. However, dissociations were noted in a minority of individuals, with some only achieving superior scores on the two face memory tests, and some only on the PMT. Performance on the Crowds test was found to be unrelated to that on the other three tasks (and may even be tapping into different cognitive processes, as indicated by the significant negative correlation for hits between the PMT and Crowds tasks).

One of the main implications of these findings regards the protocols that are currently used to detect SRs. To date, most studies have relied on performance on the CFMT+ as the sole inclusion criterion. Based on the current findings, this criterion alone would have identified 89 individuals (44.5% of the entire sample) as SRs. Yet, when tested on two related face-processing tests, consistently superior performance was only noted in 37 participants—less than half of those who would have been identified by the CFMT+ alone. This finding highlights the need for a more rigorous screening procedure that involves repeated testing. Under such enhanced protocols, individuals who are consistently accurate at face recognition across a range of tasks may be more reliably detected.

Such an approach not only provides a more rigorous inclusion criterion, but may also provide a potential means of interpreting borderline cases. For instance, while a person may, for a variety of reasons, have just missed inclusion according to performance on the CFMT+, they may subsequently score extraordinarily well on a second test of face recognition that more convincingly identifies their superior face memory skills. In the current study, 34 individuals outperformed controls only on our new test of face memory, and these individuals would have been “missed” by the CFMT+. While a strong correlation was observed between the CFMT+ and hits on the MMT, it is also important to consider the important differences in paradigm, which may have implications for real-world face recognition performance. While the CFMT+ uses tightly controlled, cropped greyscale images of faces, the MMT was designed to embrace the natural variability that occurs between images of the same person in everyday face recognition scenarios. Further, we included target-absent trials in the MMT—a condition that is not present in the CFMT+. Including target-absent responses allowed a more fine-grained analysis of the skills underpinning excellent performance. On a group level, higher accuracy appears to be driven by increases in both hits (correct identifications when the target is present) and correct rejections (when the target is not present), and is not simply related to increased response bias (e.g. increased willingness to respond “no” when uncertain). This pattern was mirrored by high performers in the PMT and the single high-performer in the Crowds test, suggesting that it is not an artefact of the procedure used in the memory task. The design of the memory task also allowed us to discriminate between correct identifications (which likely reflect actual identification of the target face) and misidentifications (which could reflect uncertainty or guessing). This analysis revealed an important distinction between superior and control performers on the MMT: the former make relatively fewer misidentification errors than the latter. In combination, a real-world interpretation of this finding is that SRs may be less likely to make incorrect identifications—both in situations where the target is present (less misidentifications) and when they are not (more correct rejections). Thus, analysis of the type of error that is typically made in a memory task may be (and arguably *should be*) an important aspect of future real-world SR screening programmes.

Another way of looking at the consistency of overall performance is to create an index across related tests. Given that the PCA dissociated performance on target-present and target-absent trials across three of the four tests, we averaged scores across the tests to create two overall indices of performance. A dissociation between performance on target-present and target-absent trials has been reported in previous work (e.g. Megreya & Burton, 2007), and held here for both the control and experimental groups. Because we found no effect of response bias on any of the tasks, it is unlikely that this factor can explain the pattern of results. Instead, it seems that different individuals may be more accurate at target-present versus target-absent judgements. Indeed, only five individuals exceeded the cut-off values for superior performance on both indices—a figure that is substantially lower than the 37 individuals who outperformed controls on overall scores for each test. Further, while 103 of the 200 members of the experimental group surpassed the control cut-off value on target-present trials, only nine individuals exceeded control performance on the target-absent trials. In part, this pattern occurred because of the larger standard deviation in control performance on target-absent compared to target-present trials, resulting in a higher cut-off value for the former. It should also be noted that the target-present index was averaged from scores on three tests, whereas the target-absent index only resulted from two test scores (because the CFMT+ only contains target-present items). These issues aside, the data do indicate dissociations between target-present and target-absent performance, with very few individuals surpassing the cut-off value on both measures. Because target-absent judgements are of fundamental importance in a policing setting (i.e. accurately deciding that a suspect is not the person in CCTV footage prevents potential miscarriages of justice or waste of police time), future SR screening should take heed of both target-absent and target-present performance. Combining these scores into overall test performance, or even in overall indices, may obscure relative weaknesses on one measure as opposed to the other.

A second implication of the current work concerns the possibility that some individuals only excel at either face memory or face matching. This hypothesis has been raised in previous work using small case series or individual case studies (e.g. Bennetts, Mole, & Bate, 2017; Bobak, Bennetts, et al., 2016; Bobak, Hancock, & Bate, 2016). While it was clear that performance on the face matching task (the PMT) was at least mildly related to the two face memory measures, the current study nevertheless identified 18 individuals who only performed in the superior range on the PMT (although the consistency of this performance was not checked in a second related task) and 13 individuals who only performed in the superior range on the face memory tasks. In many of these individuals, performance on all three tasks was nevertheless in the range that encompasses the upper end of “normal” (i.e. that above 1 SD, or even 1.5 SDs, of the control mean on all three tasks), supporting the argument that the three tasks are inter-related at least to some degree. However, for three “super matchers”, the difference between scores on the CFMT+ and the PMT were significantly larger than the mean difference between scores observed in controls. This finding provides more convincing support for a dissociation between super face matchers and super face memorisers; although it is of note that this pattern only emerged in a very small proportion of our sample, and that no evidence was observed for the reverse dissociation. That is, while superior face matching skills may be observed in the absence of superior face memory skills, people with excellent face memory skills also seem to have very good face matching skills. This finding supports hierarchical models of face-processing (e.g. Breen, Caine, & Coltheart, 2000; Bruce & Young, 1986; Ellis & Lewis, 2001), acknowledging the contribution of earlier perceptual processes in identity recognition. Such models make the assumption that perceptual analysis of a face occurs prior to identity recognition, and needs to be successfully completed in order for recognition to occur. This is backed up by the performance of those with prosopagnosia—while case studies have been reported where individuals have impairments to face memory alone, or to both facial identity perception and face memory (for a review see Bate & Bennetts, 2015), there are no reports of impaired facial identity perception in the context of intact face memory. The evidence reported here fits nicely with patterns of impairment in prosopagnosia, providing novel evidence from top performers that further bolsters the claims of theoretical models of face-processing. Importantly, then, SR screening procedures should include face matching measures from the outset, given that reliance on the CFMT+ (or any face memory measure) alone would overlook some individuals with superior face matching skills.

A similar argument may be directed towards the patterns of performance observed on the Crowds test. Using the original criterion of 1.96 SDs above the control mean, only *one* participant outperformed controls. Although the Crowds test had the greatest variability in performance of both controls and self-referred SRs, it was calibrated so that performance up to 3 SDs from the control mean could be detected (as confirmed in initial pilot-testing), and correct responses were recorded from at least some participants for every trial. It is possible that this test relies on a different set of sub-processes to the other three tests, and that successful performance relies less on the face recognition system itself. Indeed, the searching of crowds requires a range of perceptual and attentional skills that are likely not employed in face recognition tasks involving the simultaneous presentation of only two or three faces. Notably, a larger proportion of the control compared to the experimental sample performed above 1 SD from the control mean, and the top and bottom performers in the experimental group displayed varied performance on the other tests in the battery. However, given that we did not test for consistency in performance on this task, we cannot firmly reach this conclusion without further testing. Alternatively, it may be the use of composite faces that has brought about differences in performance levels.

There is good reason to suppose that this may be the case. It is inevitable that constructing a face from memory, even using a protocol designed to create identifiable images (e.g. Frowd et al., 2012), leads to inaccuracies in the resulting shape and appearance of individual features, and placement of features on the face (e.g. Frowd et al., 2005). Consequently, such composite faces are usually much harder to recognise, or even match to target, than photographs of the target identities themselves (e.g. Frowd et al., 2014; Frowd, Bruce, McIntyre, & Hancock, 2007). As mentioned earlier, EvoFIT involves a focus of construction on the internal features (e.g. Frowd et al., 2012), to coincide with the likely focus of attention for later naming using familiar face recognition (e.g. Ellis et al., 1979). However, completion of the Crowds task involves unfamiliar face perception, and so is likely to be dominated by external features, in particular hair (e.g. Bruce et al., 1999; Frowd, Skelton, Butt, Hassan, & Fields, 2011), face shape and age, so-called “cardinal” features (Ellis, 1986). Optimised in this way, it is not too surprising that the Crowds task was neither predicted by performance on the memory tasks (no reliable correlations, Table 7) nor on the PMT (reliable but *negative* correlation for both hits and CRs between the PMT and the Crowds test); indeed, low and high performance on the Crowds task led to a similar proportion of participants performing well on memory tasks and the PMT.

So, the Crowds task requires unique ability to match an error-prone stimulus (a composite) to a large number of unfamiliar face alternatives (a crowd of people). Indeed, the process involved with other holistic systems—EFIT-V or EFIT-6 (Gibson, Solomon, Maylin, & Clark, 2009) and ID (Tredoux, Nunez, Oxtoby, & Prag, 2006)—is somewhat similar to EvoFIT, resulting in an error-prone face, and so one would anticipate our results to generalise to other implementations. It is conceivable, however, that familiarity with composite stimuli in general may actually be beneficial. If this is the case, a randomly selected sample of police officers who are used to viewing facial-composite images would be expected to outperform our controls on this task. While further research is clearly needed to explore the precise underpinnings of successful performance, and indeed whether the test successfully mimics the intended real-world scenario, it may be tentatively inferred that some very specific real-world face-processing tasks require the recruitment of a different set of individuals. Regardless of whether the top performers will be those with natural facilitations in more general skills or those with experience with artificial facial images, screening for superior performers on some real-world tasks may require targeted tests that closely resemble the scenario in question.

Finally, it is of note that the sample of participants screened in this study all contacted us in the belief that they are SRs. While 18.5% of the participants outperformed controls on any three tests in the battery, a further 41% surpassed cut-off values on any two tests. It can therefore be seen that 59.5% of the sample displayed at least some consistency in superior performance (and 51% outperforming controls on the target-present index), indicating that there is utility of self-report measures in screening. However, 55 of the 200 participants (27.5%) failed to score within the superior range on any one test, and 13% only achieved the superior range on any one test. While these individuals may be genuinely mistaken about their face recognition ability, perhaps due to their point of comparison being the relatively weaker skills of a significant other, it is possible that the tests simply failed to detect their superior skills. This may be due to their reliability (although the identification of 59.5% of the experimental sample is respectable) or that they are not tapping every process which contributes to the self-perception of superior face recognition skills. For instance, our battery of tests used facial stimuli that were cropped above the neck, whereas in everyday life other aspects of the person may facilitate recognition, such as characteristics of the body and its movements. While future work should attempt to more extensively test person (and not just face) recognition skills, it can nevertheless be concluded that subjective self-report cannot reliably be used in place of objective testing. What is perhaps more striking is that only five individuals outperformed controls on both the target-present and target-absent indices, with many more surpassing cut-off values on the former but not the latter. This may indicate that self-report is based on target-present performance, given that everyday instances of recognition are likely given more weight than successful target-absent judgements. If “true” SRs are those who are top performers on both measures, they may be much less prevalent than previously thought, and more difficult to detect via self-report.

It also remains to be seen whether random sampling can identify any potential SRs who have no self-belief that they are adept at face recognition, in which case objective screening of all available personnel in applied settings should be encouraged. This question can somewhat be addressed by examination of the control data reported here, although the sample size is not representative of a wider screening procedure. When examining the data for the CFMT+, MMT and PMT, only two controls surpassed the cut-off values on any of the test: one individual achieved a score of 95/102 on the CFMT+, and another scored 43/48 on the PMT. Neither individual scored close to the cut-off values on the other tests, nor in their combined index scores. While the sample size is too small to draw any firm conclusions about the utility of random sampling irrespective of self-belief, it may be prudent to encourage all existing personnel to participate in SR screening programmes, regardless of self-perceived face recognition ability.

## Conclusions

In sum, this paper has provided evidence to suggest that current screening protocols for super recognition need to be expanded. Both face memory and face matching skills should be assessed using both target-present and target-absent trials, but inclusion criteria should not require exceptional performance on both processes. Further, some very specified real-world face recognition tasks may require targeted screening using measures that specifically replicate the required scenario. Finally, our data indicate that the new screening measures developed in this test may be of benefit to the wider field, and the new MMT may be a particularly sensitive test for the detection of SRs. We are happy to share these resources with other researchers on request (please contact the corresponding author).

## Additional file

Additional file 1: Preparation of the composite stimuli for the Crowds matching test. (DOCX 17 kb)
