# Supplementary material for: Applied screening tests for the detection of superior face recognition
Source: Cogn Res Princ Implic. 2018 Jun 27;3:22. doi: 10.1186/s41235-018-0116-5 (PMC6019417; doi:10.1186/s41235-018-0116-5)
Supplement: Supplementary file 1 — Preparation of the composite stimuli for the Crowds matching test. (DOCX 17 kb) [file 41235_2018_116_MOESM1_ESM.docx]

**Additional file 1: Preparation of the composite stimuli for the Crowds Matching Test**

Due to the inherent error present in facial composites that have been constructed by participants in laboratory experiments (akin to real world use), we thought that the proposed task would be difficult to complete accurately using composites as probes. As such, we designed a procedure to obtain composites that should be suitable for target-present and target-absent arrays in the final task. We assumed that the composites would naturally vary in accuracy to a given target, and so created more composites than was necessary, and selected items via a similarity-rating procedure. Pilot testing was then used to select images for the final task. The design for the creation of composites for target-present arrays thus involved one group of participants constructing the composites, another group giving ratings of likeness to these composites, and a third group pilot-testing performance on the final task using a specific subset of composites. A further 40 participants enabled creation of stimuli (foils and composites) for target-absent arrays. All participants were students from Bournemouth University who were given a small financial incentive for their time.

*Creation of target-present arrays:* To create the target-present trials, 20 images were randomly selected from an overall set of 60 crowd images (purchased from online image databases, e.g. www.shutterstock.com). The images displayed crowds of Caucasian adults, mostly within the perceived age range of 20 to 40 years, and most of the faces within each image were facing the camera. An experimenter selected four potential target faces (that were Caucasian, frontal facing, and perceived to be 20-40 years of age) from each image (half male), and one face from each set of four was then randomly selected for use in the study. The final 20 images were cropped and enlarged (to dimensions of 9.5 x 9.5 cm) so that the entire face (including the external features) was displayed from the neck upwards, and were printed in colour.

*Creation of composites:* Eighty participants (40 female) aged 18-39 years (M = 22.8 years, SD = 4.5) individually created a single composite image of one of the 20 target faces, resulting in four composites per target. Two male and two female participants were allocated to each target face. Participants worked with a single experimenter throughout. They viewed a print-out of a target face for 30 seconds, randomly selected, and were instructed to memorize it. Following the procedure of Fodarella, Kuivaniemi-Smith, Gawrylowicz and Frowd (2015), the experimenter then informed the participant of the overall procedure used to construct the face with EvoFIT. This explanation lasted for approximately two minutes, which also acted as a filler task between presentation of the target face and commencement of face construction.

The procedure to construct a face using EvoFIT is somewhat involved, and has been detailed elsewhere (see Fodarella et al., 2015). For the sake of brevity, we provide a summary here. Participants were asked to think back, visualise their target face and freely recall it in as much detail as possible without guessing; the experimenter wrote down information recalled on a face-description sheet (an A4 sheet of paper with feature description labels). After selecting a database appropriate to the age and gender of the target face, the experimenter presented screens showing the central (internal features) region of faces for participants to select for best overall match of the target. Selected choices were combined and the selection procedure was repeated. Next, participants were asked to select the best-matching item and enhance it using 'holistic' tools, to improve the age, weight and other overall aspects of the face; and a 'shape' tool, to improve the size and position of facial features. Participants were invited to select the best-matching set of external features (hair, ears and neck) and were encouraged to enhance the face further using holistic and shape tools. The composite image was constructed in about an hour.

*Selection of composite stimuli:* To select the final composites for the test itself, a new set of 20 participants (10 male, M age = 26.8 years, SD = 4.9) took part in a similarity-rating task. The 80 composites were each paired with their relevant target face (i.e. four composites were paired with each of the 20 targets), and participants viewed each pair (composite image size: 9.5 cm x 6.8 cm; target image: 9.5 cm x 9.5 cm) for an unlimited time in a different random order for each person. They were required to make a similarity judgment for the pairings using a Likert scale that ranged from 1 (not at all similar) to 5 (very similar).

The mean similarity rating for each pairing was calculated across participants, and we assessed the performance of composites that had received the highest rating for each target face. This approach aimed to promote best performance. A pilot test (also including the 20 target-absent trials described below) using 10 participants (5 female, M age = 27.8 years, SD = 7.2) indicated that the trials were sufficiently difficult (overall M = 53.00% for target-present trials), and we therefore retained the most similar composites for use in the final test. However, because mean accuracy was lower than the required calibration (we were aiming for 60-70% accuracy, with scope to detect performance up to three standard deviations above the mean score), we carried out materials analyses to identify the trials that were incorrectly answered most frequently. When four trials were excluded on this basis, performance of the remaining target-present trials fell within the required range (M = 63.13%).

*Creation of target-absent arrays:* To create stimuli for target-absent trials, we followed the procedure used by Henderson, Bruce and Burton (2001). First, 20 of the remaining 40 crowd images were randomly selected for use in the final test trials, and one target face (10 male) was selected from each image. Forty foil faces (20 female) were also selected from the remaining 20 crowd images (the pictures of the crowds themselves were not displayed in any part of the final test). Twelve new participants (six male, M age = 20.9 years, SD = 2.0) were presented with colour print-outs of the 40 potential foil faces, simultaneously displayed in an array. They were given a print-out of each of the 20 target faces in turn, and asked to select five foils from the array that were most similar to each target face. The foil that was most frequently selected for each target was selected for use in the appropriate trial.

Next, 20 new participants (10 female, M age = 22.1 years, SD = 4.3 years) created a composite of one of the foil faces. The same participants that were used for the similarity task described above also completed the same task for target-absent pairings. They viewed each composite in two different pairs, once with the target face and once with the selected foil face. The mean similarity ratings for both sets of faces did not differ from those collected for the target-present composite faces (*p*s > .05). The target-absent trials were subsequently created, and calibration was pilot-tested by the same participant group as for the target-present trials. Again, because performance was lower than required (55.00% for target-absent trials), materials-analyses identified the hardest four trials, and these were removed to provide a mean accuracy of 62.50% on the final 16 target-absent trials.
